# Action mechanisms and research methods of tRNA-derived small RNAs

**DOI:** 10.1038/s41392-020-00217-4

**Published:** 2020-06-30

**Authors:** Yaoyao Xie, Lipeng Yao, Xiuchong Yu, Yao Ruan, Zhe Li, Junming Guo

**Affiliations:** 1 Department of Biochemistry and Molecular Biology, and Zhejiang Key Laboratory of Pathophysiology, Medical School of Ningbo University, 315211 Ningbo, China; 2 Ningbo College of Health Sciences, Ningbo, 315000 Zhejiang China

**Keywords:** Non-coding RNAs, Molecular medicine

## Abstract

tRNA-derived small RNAs (tsRNAs), including tRNA-derived fragments (tRFs) and tRNA halves (tiRNAs), are small regulatory RNAs processed from mature tRNAs or precursor tRNAs. tRFs and tiRNAs play biological roles through a variety of mechanisms by interacting with proteins or mRNA, inhibiting translation, and regulating gene expression, the cell cycle, and chromatin and epigenetic modifications. The establishment and application of research technologies are important in understanding the biological roles of tRFs and tiRNAs. To study the molecular mechanisms of tRFs and tiRNAs, researchers have used a variety of bioinformatics and molecular biology methods, such as microarray analysis, real-time quantitative reverse transcription-polymerase chain reaction (qRT-PCR); Northern blotting; RNA sequencing (RNA-seq); cross-linking, ligation and sequencing of hybrids (CLASH); and photoactivatable-ribonucleoside-enhanced cross-linking and immunoprecipitation (PAR-CLIP). This paper summarizes the classification, action mechanisms, and roles of tRFs and tiRNAs in human diseases and the related signal transduction pathways, targeted therapies, databases, and research methods associated with them.

## Introduction

Transfer RNAs (tRNAs) have been among the focuses of biomedical researchers since they were discovered by Zamecnik and Hoagland more than 50 years ago. Using RNA polymerase III (RNA Pol III), tRNA genes can be transcribed into precursor tRNAs (pre–tRNAs), each with a 5′ leader sequence and a 3′ tail region. Subsequently, RNase P and RNase Z remove the leader sequence at the 5′ end and the trailer sequence at the 3′ end, respectively; nucleotide transferase adds the “CCA” sequence to the 3′ end; and then, through posttranscriptional modification, the sequence is folded into the secondary clover structure of mature tRNA. The tRNA clover structure consists of a dihydrouracil loop, dihydrouracil arm, anticodon loop, an anticodon arm, variable loop, pseudouracil loop (TψC loop or T loop), pseudouracil arm, and amino acid arm. tRNAs are the core components of the intracellular translation machine. The activated aminoacyl tRNA can accurately transport amino acids to the peptide chain being synthesized, and thus plays an indispensable role in the translation of genetic information.

In recent years, an increasing number of studies have found that mature tRNAs or pre-tRNAs are specifically sheared to tRNA-derived small RNAs (tsRNAs), tRNA-derived fragments (tDRs, including tRFs) and tRNA halves (tiRNAs).^1,2^ Our understanding of tRFs and tiRNAs is growing increasingly deep; they have various biological functions: acting as microRNAs (miRNAs) and regulating translation, gene expression, cellular stress response, etc.^1–3^ This paper outlines the action mechanisms, roles in human disease, related signal transduction pathways and targeted therapies of tRFs and tiRNAs, and the research methods used to study them.

**Fig. 1:**
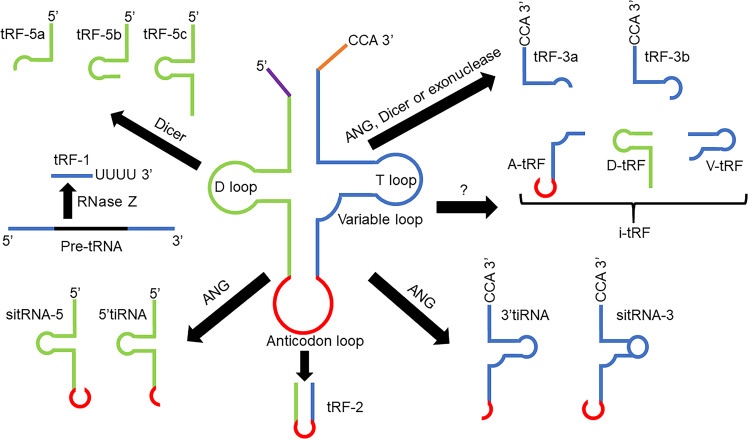
Categorization of tRNA-derived fragments (tRFs) and tRNA halves (tiRNAs). tRFs are classified into five subclasses, i-tRF, tRF-1, tRF-2, tRF-3, and tRF-5. i-tRF, tRF-2, tRF-3, and tRF-5 are derived from mature tRNAs digested by Angiogenin (ANG), Dicer, or other RNases at different sites, while tRF-1 is derived from pre-tRNA digested by RNase Z. tiRNAs are divided into two major subtypes, 5′tiRNA and 3′tiRNA, which are derived from mature tRNAs cleaved by ANG at the anticodon ring. Fragments are longer than tiRNAs are called stress-induced tRNA-5 (sitRNA-5) or sitRNA-3

tRFs are approximately 14 nucleotides (nt)−30 nt in length. Since these tRFs are similar in length to miRNAs and have 5′-phosphate and 3′-hydroxyl groups, they have gradually attracted attention over the years.^4^ Based on their biogenesis and relative length, tRFs are grouped into five major subclasses, i-tRF, tRF-1, tRF-2, tRF-3, and tRF-5 (Fig. 1). Originating from the 5′ end of the mature tRNA, the production of tRF-5s relies on Dicer cutting the D-loop or the arm stem between the anticodon loop and D-loop.^5^ Depending on the cleavage sites, tRF-5 is grouped into one of three subtypes: tRF-5a (14 nt–16 nt), tRF-5b (22 nt–24 nt), and tRF-5c (28 nt–30 nt).^6^ Among these subtypes, tRF-5a is generated by cutting the D-loop, while tRF-5b and tRF-5c are formed by cutting the D-stem and anticodon stem, respectively. tRF-3, generated from the 3′ end of mature tRNA, is digested by angiogenin (ANG), Dicer or exonuclease at the TψC loop. Consequently, in the tRF-3 tail, there is a “CCA” trinucleotide that is specific to the 3′ end of this mature tRNA. The subtypes of tRF-3 are tRF-3a (18 nt) and tRF-3b (22 nt).^6^ Some researchers have shown that tRF-3 and tRF-5 are generated in a Dicer-dependent fashion;^5^ however, a recent study reported that the biological origin of tRF-3 and tRF-5 might not be dependent on Dicer.^7^ These tRF subclasses can be found in small RNA data sets in all types of analyses, from yeast to human.^6^ tRF-2 is produced by the decomposition of the anticodon loop of tRNA under hypoxic conditions and do not include the typical 5′ end and 3′ end groups.^8^ tRF-1, also called 3′U-tRF, originates at the 3′ untranslated regions (UTR) of pre-tRNA and is digested by RNase Z with the characteristic of the poly-U sequence.^9^ i-tRF originates from the internal zone of any mature tRNAs, but not the 5′-terminal and 3′-terminal regions. i-tRF is named based on the starting position of the 5′ end in the tRNA. Among the i-tRFs, A-tRF and V-tRF represent fragments generated by cuts at the anticodon ring and variable area, while D-tRF are fragments formed by cuts at the D stem.^10^ Each tRF may have its own four-digit code or other form of identification (ID).

tiRNAs are produced by cleaving the anticodon ring of the mature tRNA (Fig. 1). tiRNAs are produced under stress conditions such as phosphate deficiency, lack of amino acids, heat shock, ultraviolet radiation, hypoxia, oxidative stress, and viral infections.^11^ According to whether the 5′- or 3′-sequence contains the anticodon cut locus, tiRNAs are divided into two subtypes, 5′tiRNA and 3′tiRNA, with lengths of 31 nt–40 nt.^12^ Among these two types, 5′ tiRNAs begin at the 5′ end of the mature tRNA and end at the anticodon ring, while 3′ tiRNAs include the anticodon ring and the 3′ end.^13^ ANG is mainly involved in the production of tiRNAs in mammals, and ANG-mediated tiRNAs are precisely regulated.^14^ In a recent study, Su et al. found that ANG is not the only RNase that produces tiRNAs; other RNases can produce tiRNAs.^15^ Researchers observed fragments in *Giardia lamblia* of ~46 nt in length, which were longer than tiRNAs.^16^ The 5′ fragment of mature tRNA is named stress-induced tRNA-5 (sitRNA-5). Similarly, the 3′ fragment of tRNA is named sitRNA-3.^16^

## Mechanisms of tRFs and tiRNAs

tRFs and tiRNAs play biological roles through a variety of mechanisms, including interactions with proteins or mRNA, regulation of gene expression, control of the cell cycle, and regulation of chromatin and epigenetic modifications. In addition, Torres et al. found that differential expression of tRNA genes results in changes in the abundance of tRFs not the abundance of mature tRNA.^17^ This finding means that differences in tRNA gene expression modulate the ability of tRFs to perform noncanonical tRNA functions.^17^

### Interacting with proteins or mRNAs

**Fig. 2:**
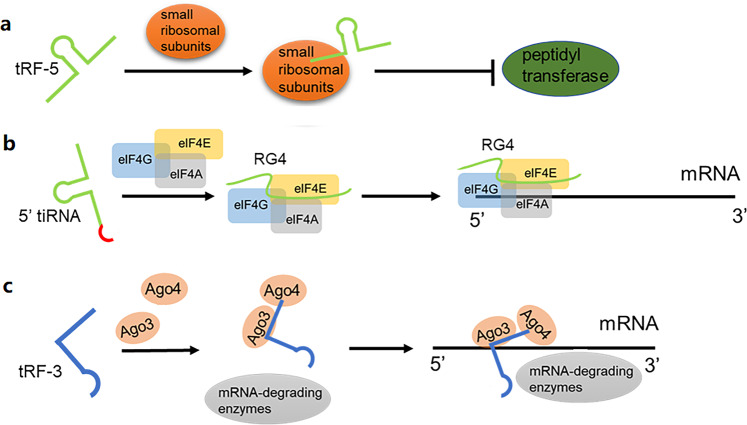
tRFs and tiRNAs regulate translation. **a** By binding to small ribosomal subunits, tRF inhibits peptidyl transferase activity and leads to weakened translation. **b** 5′tiRNA inhibits translation by forming a RNA G-quadruplex (RG4s) that replaces the translation initiation complex eIF4G/eIF4E on the mRNA cap. **c** The combination of tRF-3 with argonaute 3 (Ago3) and Ago4 binding to mRNA allows mRNA-degrading enzymes to degrade the target mRNA

tRFs and tiRNAs inhibit translation in a variety of ways. tRFs and tiRNAs might suppress protein translation by impacting the formation of ribosomes with prolonged activity.^18^ Gebetsberger et al. found that, under specific stress conditions, *Haloferax volcanii*, a halophilic archaea, generates a tRF from the 5′ fragment of tRNA^Val5^,.^19^ Upon binding to the small ribosomal subunit, this tRF inhibits peptidyl transferase activity and weakens translation (Fig. 2a).^19^ Lalande et al. found that tRFs might be potential regulators in plant translation, such as in *Arabidopsis*, due to their ability to bind to polyribosomes.^20^ It has been found that 5′tiRNA^Ala^ and 5′tiRNA^Cys^ containing a terminal oligo-G motif (TOG motif) inhibits translation by forming an intermolecular RNA G-quadruplexes (RG4), replacing the translational initiation complex eIF4G/eIF4E on the mRNA cap (m^7^GTP) structure (Fig. 2b).^21,22^ Furthermore, researchers reported that the binding of the TOG motif-5′tiRNA to Y box-binding protein 1 (YBX1) promoted the accumulation of stress granules (SGs), “isolated” translation initiation factors from ribosomes, and further increased the overall inhibitory effect of translation.^23^

PUS7-mediated pseudouridylation (Ψ) of tRF-5 can inhibit stem cell translation. Guzzi et al. found that inactivation of PUS7 in embryonic stem cells (ES cells) weakened tRF-mediated translational regulation, resulting in embryo-specific defects and increased protein biosynthesis.^24^ It has been reported that tRF-5^Glu-CTC^, specifically induced by respiratory syncytial virus (RSV), may promote RSV replication and proliferation in human airway epithelial cells by reducing the expression of defense genes against RSV or by targeting the mRNA encoding the RSV protein.^25^ The study found that a silencing complex, formed by the combination of tRF-3 with argonaute 3 (Ago3) and Ago4, directly binds the mRNA of the target gene, allowing it to enter the specific cytoplasmic processing body with a large number of mRNA-degrading enzymes and eventually restraining the translation of the target gene by degrading the mRNA of the target gene (Fig. 2c).^26^

On the other hand, some tRFs and miRNAs have similar functions in inhibiting mRNA translation. For example, it has been found that a 3-tRF derived from tRNA^Leu-CAG^ in non-small cell lung cancer (NSCLC) cells has a similar role to miRNA, which can weaken protein translation.^27^ Researchers found significant associations between the identified tRFs and miRNAs involved in developmental processes in 32 types of cancer.^28^ Londin et al. found that, in uveal melanoma, the abundance of miRNA isoforms and tRFs was related to a variety of molecular phenotypes, metastasis, and patient survival rates.^29^ Further studies have shown that, according to the newly added miRBase database, miR-1247a and miR-1247b correspond to tRNA^Lys3^ and tRNA^Lys5^, respectively,^30,31^ and the proportion of these two miRNAs and their corresponding tRNAs shows a significant positive correlation, suggesting that these two miRNAs likely correspond to these tRFs.^32,33^ Researchers have found that pre-tRNA^Ile-UAU^ is a precursor of miRNA, which can be transferred into the cytoplasm during Dicer and Ago loading.^34^

It has been reported that several tRFs and tiRNAs affect the stability of mRNA. YBX1, a multifunctional RNA-binding protein (RBP), has multiple interacting ligands that participate in a variety of cellular signal transduction pathways. It is highly expressed in cancer cells and can bind to oncogenic transcripts to regulate tumor development. Goodarzi et al. found that, when exposed to low oxygen levels, breast cancer cells generate specific tRFs (such as tRF^Asp-GTC^ and tRF^Gly-TCC^).^35^ These tRFs competitively bind to YBX1 during oncogenic transcription. By replacing the 3′UTR in YBX1, the stability of endogenous oncogene transcripts is reduced, thereby suppressing their expression, antagonizing the activity of YBX1, and ultimately inhibiting the proliferation of tumor cells.^35^ In addition, these tRFs can induce the formation of SGs in the cytoplasm. The assembly of SGs temporarily silences mRNA in a cell, allowing energy to accumulate for use by transcription- and survival-related proteins, which is beneficial to mammalian cell survival under adverse conditions.^36^ The tRFs generated under specific stress conditions can compete with siRNAs and dsRNA precursors and compete with Dicer and Ago2 without forming a new silencing complex, which functionally attenuates the inhibition of siRNAs on various types of mRNAs.^37,38^ Since Ago2 is considered a translation enhancer,^39^ it is speculated that tRFs act as a switch to trigger the function of Ago2 under specific cellular stress conditions.

### Regulating gene expression

**Fig. 3:**
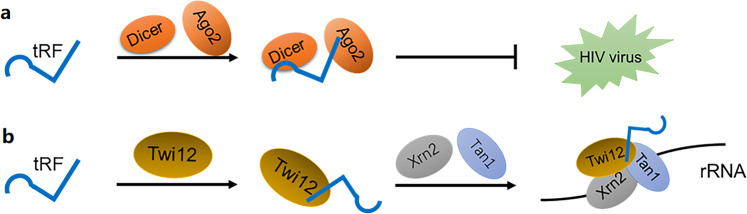
tRFs and tiRNAs regulate gene expression. **a** tRF promotes HIV suppression by acting as a silent reporter by combining with Dicer and argonaute 2 (Ago2). **b** The tRF-Twi12 complex works synergistically with Xrn2 and Tan1 to regulate rRNA processing

tRFs and tiRNAs are involved in regulating gene expression. Torres reported that the expression of tRNA genes is tissue-specific with cell-type characteristics and can regulate the abundance of tRFs.^40^ In addition, Torres found that approximately one-half of human tRNA genes show silenced or low expression.^40^ Ago and Dicer are known to be essential factors participating in the regulation of gene silencing during RNA interference.^41^ A study reported that tRF-3006, comprising 18 nt, expressed in high abundance and derived from tRNA^Lys^ was found in HIV-1-infected cells.^42^ HIV RNA hybridizes with the 3′ end of tRNA^Lys^ in host cells to form double-stranded RNA; tRNA^Lys^ is reverse transcribed into cDNA by a reverse transcriptase and used as a primer for the synthesis of complementary DNA.^42^ In other words, tRF-3006 combines with 56 nt-67 nt of tRNA^Lys^ in human cells, which is the primer-binding locus of HIV genomic RNA, thus acting as a primer in the process of reverse transcription.^42^ Moreover, tRF-3006 promotes HIV suppression by acting as a silent reporter and combining with Dicer and Ago2 (Fig. 3a).^42^ It has been reported that, originating from the pre-tRNA tail region, tRF regulates viral gene expression by isolating La/SSB in the cytoplasm.^43^ Some researchers conducted Ago-immunoprecipitation (Ago-IP) tRF analysis and, using bioinformatics methods, found that tRF-5 mainly exists in multiple Ago-IPs in addition to Ago2-IP, while Ago2-IP has the strongest interaction with tRF-3.^44^ Since only Ago2 in mammals contains a slicer domain, Daugaard et al. speculated that the interaction of Ago2-tRF-3 might suppress the antisense reporter genes by directly cutting the target transcripts.^44^ Jehn et al. found that, in the primate hippocampus, 5′tiRNAs silence genes through a sequence-specific process, with the most efficient target loci aligned with the intermediate region of the 5′tiRNAs, corresponding to the target 3′UTR.^45^

tRF-3s and tRF-5s may regulate gene expression by interacting with PIWI proteins and Ago proteins.^46^ The tRF produced by tRNA^Gly-GCC^ in sperm has been found to inhibit the expression of nearly 70 genes-related to the endogenous reverse transcription factor MERVL- associated with embryos and ES cells.^47^ Upon protein restriction in mice, the level of small RNA (sRNA) in mature sperm is affected, and the level of let-7 is reduced, while the number of 5′ fragments of tRNA^Gly^ is increased.^47^ In ES cells and embryos, the tRF-5 produced by tRNA^Gly-GCC^ functionally inhibits the expression of genes-related to endogenous reverse transcription.^47^ In addition, the tendency of tRF-5s to bind to Ago1, not Ago2, is relevant to posttranscriptional RNA silencing.^48^ The Piwi protein Twi12 of tetrahymena interacts with tRF-3. The tRF-3-Twi12 complex functions synergistically with Xrn2 and Tan1 to regulate rRNA processing (Fig. 3b).^4,49,50^ Ago4-containing siRNA is translocated to the nucleus. tRFs, which are located in the cytoplasm, bind to Ago4 and may be translocated to the nucleus, where they play roles in transcription or posttranscriptional gene silencing.^51,52^

According to a report, tRNA^Glu^-derived piRNA [td-piR (Glu)] and PIWI-interacting RNA (piRNA) are more highly expressed in human monocytes than in dendritic cells.^53^ Regulation of RNA Pol III activity enables IL-4 to effectively reduce the production of tRNA^Glu^ and subsequent td-piR (Glu).^53^ In addition, the authors found that the td-piR (Glu)/PIWIL4 complex binds heterochromatin protein 1 at the CD1A promoter region, suppressor of variegation 3–9 homolog 1 (SUV39H1) and SET domain bifurcated 1 (SETDB1), which promotes the methylation of H3K9 to ultimately and significantly inhibit the transcription of CD1A.^53^

### Regulating epigenetic modifications

Several tRFs and tiRNAs have been found to act as epigenetic regulators. A father’s diet may affect the metabolism of his offspring. One study found that offspring of male mice fed high-fat diet (HFD) developed insulin resistance and impaired glucose tolerance 7 weeks after birth, which became severe at 15 weeks.^54^ The phenomenon in this mouse model was caused by 30 nt–34 nt tRFs.^54^ A whole-genome comparison between the offspring from paternal mice fed a normal diet and those fed a HFD revealed that the expression levels of genes-related to carbohydrate, ketone, and monosaccharide metabolism in the offspring of the HFD-fed paternal parent were significantly reduced.^54^ In addition, by injecting tRFs and tiRNAs from the sperm of HFD-fed mice into normal fertilized eggs, the expression profiles of the genes-related to metabolic pathways in early embryonic and islet cells changed significantly.^54^ This study confirms the effect of diet on sperm tRFs.

Sperm have a variety of genetic materials that can serve as carriers of genetic materials and thus pass genetic information to future generations. Another study of mice on a low-protein (LP) diet confirmed that tRFs play key roles in mammalian sperm maturation and fertilization.^47^ The results suggested that there was no correlation between the sRNAs of immature sperm in mouse testicles and the LP diet, but the sRNAs of mature sperm in the epididymis were significantly affected, especially at the of tRNA^Gly-GCC^ level, which was significantly increased.^47^ Further research found that the tRF produced by tRNA^Gly-GCC^ in sperm inhibited the expression of nearly 70 genes-related to the endogenous reverse transcription factor MERVL-associated with embryos and ES cells. Due to the protein restriction in the mice, the level of sRNAs in mature sperm was affected, while the number of 5′ fragments of tRNA^Gly^ was increased.^47^ In ES cells and embryos, tRF-5 produced by tRNA^Gly-GCC^ functionally inhibited the expression of genes-related to endogenous reverse transcription.^47^ These studies have confirmed the effect of diet on mouse sperm tRFs. In addition, tRF^Glu-TTC^ has been reported to be a potential new epigenetic regulator of fat production.^55^ tRFs in sperm with epigenetic factors from a parent can alter the expression of some offspring genes and cause corresponding metabolic disorders.

### Regulating the cell cycle

tRFs and tiRNAs regulate cell proliferation by participating in the cell cycle process. tRF⁃1001, a type of tRF-1 from the 3′ end of precursor tRNA^Ser^, is necessary for tumor cell growth.^56^ Knocking down tRF-1001 can interfere with cell proliferation, arrest cells in the G2 phase, and inhibit DNA biosynthesis.^57^ Moreover, Hauseecker et al. found a specific correlation between tRF-1001 and Ago proteins, especially Ago3 and Ago4.^58^

It has been reported that some tRFs and tiRNAs bind to cytochrome C and inhibit its binding with Apaf-1 and then prevents the activation of caspase-9, which inhibits the formation of apoptotic bodies, thereby inhibiting apoptosis.^59,60^ In normal cells, tRFs act as endogenous apoptotic signals to suppress the regulators of relevant apoptotic proteins, directly or indirectly causing apoptosis. When cells are stressed, tRFs increase significantly, causing apoptotic processes to escape regulation and simultaneously inducing malignant cell proliferation.^61^ In addition, TRMT10A (a tRNA methyltransferase) deficiency mediated by the 5′tRNA^Gln^ fragment induces pancreatic β-cell death.^62^

### Regulating chromatin

tRFs and tiRNAs are relevant to the regulation of chromatin. The nuclear localization of tRF-5 and its relationship with Ago proteins suggest that some tRFs may act in chromatin regulation.^63^ CU1276, a tRF dependent on Dicer1 enzymatic shearing, participates in the regulation of B-lymphocyte proliferation by acting as an miRNA; in lymphoma, downregulating the expression of CU1276 can alleviate DNA damage, thereby reducing the accumulation of tumor mutants and improving the tolerance of cells to chromosomal aberrations.^64^

## Roles of tRFs and tiRNAs in human diseases

### Cancers

Recently, researchers have revealed the tsRNA signature in a variety of cancers, including chronic lymphocytic leukemia (CLL), lung cancer, colon cancer, prostate cancer, breast cancer and ovarian cancer, through gene chip technology and found that the activation of oncogenes and the inactivation of tumor suppressors led to the abnormal expression of certain specific tRFs and tiRNAs in various cancers.^65^

In B-cell lymphoma, researchers found that some tRFs inhibited cell proliferation and regulated the DNA damage response by modulating the expression of DNA damage response genes.^64^ Studying myelodysplastic syndromes (MDS), Guo et al. reported that the expression levels of tRFs in samples before treatment might predict the response of DNA methyltransferase inhibitor therapy.^66^ Compared with MDS patients who had never progressed to acute myeloid leukemia (AML), the expression level of tRF^Asp^ in MDS patients who later developed AML was significantly reduced. tRF^Asp^ may be a biomarker for predicting the progression from MDS to AML.^67^ There is evidence of mutations at the ts-101 and ts-53 gene loci in CLL and lung cancer cells, suggesting that these tsRNAs may serve as biomarkers for cancer diagnosis and treatment.^46,68^

Sun et al. measured the expression levels of tRFs in normal breast epithelial cell lines and trastuzumab-sensitive and trastuzumab-resistant breast cancer cell lines, and the results showed that tRFs were differentially expressed in different cell lines, and the overexpression of tRF-30JZOYJE22RR33 and tRF-27-ZDXPHO53KSN can be regarded as independent predictors of progression-free survival of patients with breast cancer.^69^ Therefore, tRF-30-JZOYJE22RR33 and tRF27-ZDXPHO53KSN may be potential biomarkers and intervention targets for the clinical treatment of trastuzumab-resistant breast cancer. In triple-negative breast cancer (TNBC) cell lines under hypoxic conditions, the expression levels of tDR-0009 (derived from tRNA^Gly-GCC-1-1^) and tDR-7336 (derived from tRNA^Gly-GCC-1-2^) were significantly increased.^70^ These two tRFs are mainly related to the maintenance of stem cell populations and the response to IL-6, which may be the mechanisms by which doxorubicin resistance is promoted in TNBC. A study has shown that the abundance of tRNA^His-GTG^, tRNA^Gln-TTG^, and tRNA^Gln-CTG^ in TNBC cells was significantly lower than that of normal breast cells, and the specific tRNA sites (such as nuclear tRNA^Gly^ and tRNA^Leu^ and mitochondrial tRNA^Val^ and tRNA^Pro^) were closely related to ethnic differences in TNBC.^71^ Researchers have found that runt-related transcription factor 1 (RUNX1) may prevent the excessive malignant proliferation of breast cancer epithelial cells by inhibiting the carcinogenesis of ts-112, thereby enhancing its role in maintaining the healthy function of breast epithelial cells.^72^

Researchers have used RNA-seq to analyze the expression levels of tRF in normal prostate tissues adjacent to prostate cancer tissues and with cells from tissues at different stages of prostate cancer, and found 598 differentially expressed tRFs.^73^ In recurrent prostate cancer, the tRF from tRNA^Phe-GAA^ was significantly downregulated compared with that of the adjacent normal tissue, while the tRF from tRNA^Lys-CTT^ was upregulated and expressed at higher levels in higher grade prostate cancer, and the patients with highly expressed tRF based on tRNA^Lys-CTT^/tRNA^Phe-GAA^ had shorter survival and recurrence periods.^27^ Therefore, the tRF ratio tRNA^Lys-CTT^/tRNA^Phe-GAA^ may be a meaningful marker of prostate cancer progression. tRF^Leu-CAG^ has a clear correlation with the stage of lung cancer, and repressing the expression of tRF^Leu-CAG^ can inhibit cell proliferation and prevent cell-cycle progression.^27^ In ovarian cancer, tRF-5, produced by tRNA^Glu-CTC^, binds to the 3′UTR site of breast cancer anti-estrogen resistance 3 (BCAR3) mRNA, which suppresses the expression of BCAR3, thereby inhibiting the proliferation of ovarian cancer cells.^74^

### Other human diseases

Some studies of the nervous system have shown that ANG can promote the accumulation of tiRNAs due to defects in tRNA methyltransferases Dnmt2 and NSun2, thereby causing the stress response of and cell death in the nervous system, which indicates that tiRNAs build up to cause neuronal death.^75,76^ However, tiRNAs (e.g., tiRNA^Ala^ and tiRNA^Cys^) or their DNA analog with G4-motif structures have been found to facilitate the survival of neurons under stress conditions, which may contribute to the treatment of neurodegenerative diseases.^77,78^ In addition, tRFs and tiRNAs are also associated with infectious diseases. For instance, *Trypanosoma cruzi* excretes tRFs and tiRNAs extracellularly, which are then transferred to other parasites and mammals, resulting in metacyclogenesis transformation and augmenting the susceptibility to infection.^79^ Other researchers found that, in the case of tissue injury, such as ischemia-reperfusion, radiation, and toxic damage, the expression levels of tRFs and tiRNAs are correlated with the degree of tissue injury.^80^

## tRF- and tiRNA-related signal transduction pathways and targeted therapies

Wang et al. have reported that the target function of tiRNA^Tyr-GTA^ in colorectal cancer (CRC) is mostly concentrated in the negative feedback regulation of epithelial cell apoptosis and peroxisome proliferator-activated receptor signaling pathway.^81^ In addition, tRF/miR-1280 suppresses the growth and metastasis of CRC by inhibiting the Notch signaling pathway that maintains the function of cancer stem cell-like cells.^82^ Moreover, in CRC, several differentially expressed mRNAs are potential targets of differentially expressed tRFs and key miRNAs, which mainly act on the vitamin-containing metabolic pathway and the cyclic guanine monophosphate/protein kinase G signaling pathway.^83^ These results may contribute to the prediction and treatment of CRC. In lung cancer, tRF^Leu-CAG^ interacts with the AURKA protein and modulates the Wnt/β-catenin and PI3K/Akt signaling pathways to alter histone proteins, thereby inducing the epithelial-to-mesenchymal transition.^27,84^ Compared with normal controls, 101 tRNAs and 355 tsRNAs are significantly differentially expressed in systemic lupus erythematosus (SLE).^85^ A Kyoto Encyclopedia of Genes and Genomes pathway analysis showed that the change in expression of genes downstream of tRNAs are the most abundant in SLE and that the change in the expression of genes downstream of tsRNAs are the most abundant in the T-cell receptor signaling pathway, Th1 and Th2 cell differentiation, and primary immunodeficiency.^85^ These mechanisms may be involved in the occurrence of SLE. Overall, tRFs and tiRNAs are very closely related to the occurrence and development of human diseases and may be potential biomarkers for diagnosis and clinical treatment targets.

## Methods for studying tRFs and tiRNAs

To study the molecular mechanisms of tRFs and tiRNAs, researchers have used various bioinformatics and molecular biology methods. Currently, many technologies, including microarray analysis, real-time quantitative reverse transcription-polymerase chain reaction (qRT-PCR), Northern blotting, RNA sequencing (RNA-seq), and other technologies, are widely used in tRF and tiRNA research fields.

### tRF and tiRNA databases

tRFs were originally considered randomly degraded fragments of tRNA. However, researchers found repeated readings of tRFs that matched particular domains of mature tRNAs not random permutations corresponding to the primary structure of mature tRNAs, suggesting that tRFs may be nonrandomly biologically degraded fragments.^32^ However, since tRFs are very small (14 nt–30 nt), a method to distinguish genuine tRFs from randomly degraded products of tRNAs is the first need that should be addressed.

To facilitate research and academic exchanges, researchers have established multiple tRF and tiRNA databases (Table 1).

**Table 1:** tRF and tiRNA databases

Name	Characteristics	Website
BBcancer	Includes data on the expression of various RNA types, including tRFs, in 5040 normal and cancer blood samples from 15 cancer types.	http://bbcancer.renlab.org/
DASHR v2.0	This was the first database to integrate human sncRNA genes with mature product profiles from RNA-seq.	https://lisanwanglab.org/DASHRv2
GtRNAdb	The most frequently cited source of genetic information for web-based tRNA, data can be retrieved using sequences or genetic characteristics.	http://gtrnadb.ucsc.edu/
MINTbase	Provides easy access to information about the maximum abundance of an tRF, its specific data, and information on its parent tRNA modifications.	http://cm.jefferson.edu/MINTbase/
MINTmap	Identifies and quantifies tRFs by mining deep-sequencing data, and calculates the original and normalized abundance of an tRF.	https://github.com/TJU-CMC-Org/MINTmap/
PtRFdb	Very convenient for users to verify and further understand the characteristics of plants.	www.nipgr.res.in/PtRFdb
RNA FRABASE	Allows users to autonomously search for the required three-dimensional fragments within the RNA structure.	http://rnafrabase.cs.put.poznan.pl
SPORTS1.0	Optimized for tsRNA from sRNA-seq data and relies on nucleotide mismatches in sRNA to predict potential RNA modification domains.	https://elkssl99cd2175108d157588c04758296d1cfclib.link.nbu.edu.cn:8443/junchaoshi/sports1.0
tDRmapper	Supplies a standardized naming method and quantitative scheme for tRFs and facilitates discovery of the biological functions of tRFs.	https://github.com/sararselitsky/tDRmapper
tRF2Cancer	Accurately recognizes tRFs and evaluates their expression in a variety of cancers.	http://rna.sysu.edu.cn/tRFfinder/
tRFdb	The first tRF database; the retrieval tRF sequences may have originated from the tRNA genome coordinates and names.	http://genome.bioch.virginia.edu/ trfdb/
tRFexplorer	Allows researchers to study the potential biological effects of tRF without any direct experimental evidence.	https://trfexplorer.cloud/

tRFdb was the first tRF database and contains the tRF sequences of multiple species, including humans, and can be searched by tRF sequence or tRF ID.^86^ The advantages of this database are that the retrieved tRF sequence may have originated from the tRNA genome coordinates and names, and the corresponding sRNA libraries have tRF and parent tRNA sequence links and read count links. PtRFdb, developed by Gupta et al., is a database for plant tRFs.^87^ Users of PtRFdb can quite conveniently verify and gain understanding of the characteristics of plants. Another database, called tRFexplorer, provides investigators with the expression profiles of the ncRNA derived from tRNA for every The Cancer Genome Atlas tumor type and for each cell line in NCI-60.^55^ The advantage of this database is that it allows researchers to study the potential biological effects of an tRF without any direct experimental evidence. MINTbase v2.0 contains information on nuclear and mitochondrial tRFs from a variety of human tissues.^88^ Information about the maximum abundance of tRF and specific data and information on the parent tRNA modifications can be easily queried.^88^ Loher et al. developed Mitochondrial and Nuclear TRF mapping (MINTmap), a tool that can be used to identify and quantify tRF by mining of deep-sequencing data, can calculate the original and normalized abundance of RFs.^89^ Zheng et al. explored a web server called tRF2Cancer, which can accurately recognize tRFs and evaluate their expression in several types of cancers.^90^ Kuksa et al. developed DASHR v2.0, which was the first database to integrate human sncRNA genes with mature product profiles based on RNA-seq.^91^ The RNA FRABASE is a database that allows users to autonomously search for required three-dimensional fragments within the RNA structure, thereby providing extensive opportunities for RNA research.^92^ Another database, called BBCancer, includes data on the expression of various RNA types, including tRFs, in 5040 normal and cancer blood samples of 15 cancer types, providing an effective platform for the development of blood biomarkers.^93^ There is a novel approach called tDRmapper, which not only supplies a standardized naming method and quantitative scheme for tRFs but also facilitates the discovery of the biological functions of tRFs.^94^ Qu et al. used an Illumina NextSeq instrument to detect tRFs in CD5-positive cells of relapsed and refractory diffuse large B-cell lymphoma.^95^

### Methods for studying the roles of tRFs and tiRNAs

Microarray and RNA-seq are effective tools for the high-throughput detection of tRF and tiRNA expression. Balatti et al. performed a microarray analysis of tsRNA expression profiles of CLL and lung cancer, and found that tsRNA might be an important effector in the pathogenesis of cancer-related development.^65^ According to reports, researchers used high-throughput sequencing to find that the levels of 5′tiRNAs in the serum of breast cancer patients were closely related to the pathological characteristics of the patients.^96^ Next-generation sequencing provides an unprecedented opportunity to discover and quantify various tRFs and tiRNAs.

Researchers can design specific amplification primers to verify tRFs and tiRNAs in databases through qRT-PCR.^97^ Huang et al. used qRT-PCR to verify differentially expressed tRFs in breast cancer and further analyzed their relationships in the breast cancer context.^98^ Some researchers detected the expression level of tRF^Leu-CAG^ in serum samples of patients with NSCLC via qRT-PCR and found that tRF^Leu-CAG^ was obviously upregulated in people with stage III and IV NSCLC and showed a strong correlation with the stage of NSCLC.^27^ Qin et al. verified the tsRNA-seq results using qRT-PCR to detect differentially expressed tsRNA in rats with spinal cord injury.^99^

In addition, Northern blotting can be used to determine tRF and tiRNA expression levels. Su et al. used Northern blotting to verify the high abundance of tiRNA in placenta/decidua, confirming the authenticity of small RNA-seq experimental results.^100^ Some scholars using Northern blot analysis reported that the abundance of tRNA^Val-CAC^ and tRNA^Gly-GCC^ in *Sus scrofa* was limited to the ovary and kidney.^101^ Using Northern blotting, our group verified that tiRNA-5034-GluTTC-2 was differentially expressed in gastric cancer, which provided a basis for the potential use of tiRNA-5034-GluTTC-2 as a biomarker of gastric cancer.^97^

There is an approach called cross-linking ligation and sequencing of hybrids (CLASH), which is used to identify the interaction of RNA with RNA.^8^ This method comprises three steps. First, RNAs associated with proteins such as Ago can be stabilized by cross-linking the RNAs with proteins by ultraviolet irradiation. Then, the proteins are immunoprecipitated, and the protein-related RNAs are pruned; thus, only the RNA region surrounding the protein interaction site survives. Second, by adding a ligase to the IP complex, the pruned RNAs in an individual protein molecule are connected to each other. Third, these sRNAs are sequentially extracted, reverse-transcribed, sequenced, and then localized back into the genome, which encodes identifiable RNA-RNA chimeras produced by the interaction of the two RNAs in the immunoprecipitated protein.

A biochemical method called photoactivatable-ribonucleoside-enhanced cross-linking and immunoprecipitation (PAR-CLIP) can be used to confirm the RNA that binds to particular proteins and the binding sites of the proteins on the RNA.^102^ Photoreactive ribonucleoside analogs, such as 6-thioguanosine (6-SG) and 4-thiouridine (4-SU), are integrated into the transcripts of nascent RNA in living cells. By exposing the cells to UV light at 365 nm, photoreactive nucleoside-labeled cellular RNAs is cross-linked effectively to their interacting RBPs. After the target RBP is immunoprecipitated, the trimmed RNA retains only the cross-linked and coimmunoprecipitated regions, and then, the protein is digested. The isolated RNA is reverse transcribed into a cDNA library; then, high-throughput sequencing is performed using Solexa technology. The exact location of the cross-link in the gene can be determined by sequencing the position with the cDNA mutation. When 4-SU is used, the cross-linked RNA is mutated from a thymidine to cytidine during reverse transcription. Hence, the existence of the T–C transition at multiple cloning-specific loci indicates the specific binding sites on the RNA of the protein. When 6-SG is used, the cross-linked RNA undergoes a mutation in which guanosine is replaced by adenosine during the process of reverse transcription. Hence, the G-to-A mutations at multiple cloning-specific sites indicate the specific binding sites on the RNA of the protein. Using CLASH and PAR-CLIP methods, Kumar et al. found that in human HEK293 cells, tRFs are mainly related to Ago1, Ago3, and Ago4, but not Ago2, the major effector protein of miRNA.^6^

## Perspectives

Although an increasing number of researchers have focused on tRF and tiRNA research, we see only the tip of the iceberg. A series of remaining problems needs to be solved:

1. tRFs and tiRNAs show obvious regularity compared with randomly degraded fragments of RNA, indicating that their formation occurs through specific mechanisms in the cells. However, we do not yet know the details of the generation mechanism.
2. There is no uniform system for the naming of tRFs and tiRNAs.
3. tRFs and tiRNAs exert their biological functions by regulating the translation, gene expression, mRNA stability, epigenetic, and chromatin modifications. However, current studies have been limited to a few specific tRFs and tiRNAs, and it is unclear whether tRFs and tiRNA are specifically expressed in tissue samples.
4. The potential relationship between the large number of base modifications in tRNA and the mechanism of action of tRFs and tiRNAs is currently unclear.
5. Whether tRFs and tiRNAs overlap with their parent tRNAs through some mechanism of action, or whether there is a certain correlation is unknown and further experimental demonstration is required.
6. There are few new technologies applicable to tRF and tiRNA research.

Therefore, it is necessary to establish increasingly effective research methods to systematically study the structure and mechanism of tRFs and tiRNAs. In summary, with the development of technologies, an increasing number of tRFs and tiRNAs will be recognized and identified. We will then have a better understanding of their biological roles.
